# Socioeconomic correlates of face mask use among pedestrians during the COVID-19 pandemic: An ecological study

**DOI:** 10.3389/fpubh.2022.921494

**Published:** 2022-11-16

**Authors:** Zahra Rahimi, Mohammad Javad Mohammadi, Marzieh Araban, Gholam Abbas Shirali, Bahman Cheraghian

**Affiliations:** ^1^Department of Biostatistics and Epidemiology, School of Public Health, Ahvaz Jundishapur University of Medical Sciences, Ahvaz, Iran; ^2^Department of Environmental Health Engineering, Air Pollution, and Respiratory Diseases Research Center, Ahvaz Jundishapur University of Medical Sciences, Ahvaz, Iran; ^3^Department of Health Education and Promotion, School of Public Health, Ahvaz Jundishapur University of Medical Sciences, Ahvaz, Iran; ^4^Department of Occupational Safety and Health Engineering, School of Public Health, Ahvaz Jundishapur University of Medical Sciences, Ahvaz, Iran; ^5^Department of Biostatistics and Epidemiology, School of Public Health, Ahvaz Jundishapur University of Medical Sciences, Ahvaz, Iran

**Keywords:** socioeconomic factors, face mask use, pedestrians, COVID-19, Iran

## Abstract

**Background:**

Many countries have recommended using face masks for the general population in public places to reduce the risk of COVID-19 transmission. This study aimed to assess the effects of socioeconomic status on face mask use among pedestrians during the COVID-19 pandemic.

**Methods:**

This cross-sectional study was conducted in Ahvaz, southwest Iran in August 2020. A total of 10,440 pedestrians have been studied from 92 neighborhoods of the city. Three socioeconomic indicators including Land price, Literacy rate, and the Employment rate for each neighborhood were used in this study. Analysis of Covariance and partial correlation coefficients were applied to assess the relationship between prevalence rates of mask usage and SES indicators.

**Results:**

The mean ± SD age of the pedestrians was 32.2 ± 15.1 years. Of 10,440 observed participants, 67.9% were male. The overall prevalence of face mask usage was 45.6%. The prevalence of mask usage in older people and women was significantly higher than the others. The three assessed socioeconomic indicators were directly correlated to the prevalence of mask usage at individual and neighborhood levels.

**Conclusion:**

We found that literacy had the strongest correlation with the prevalence of mask usage compared to the land price and employment rate among the three assessed SES indicators. Hence, it can be concluded that the social component of socioeconomic status has a greater effect on mask usage by people than the economic component of socioeconomic status.

## Introduction

The novel coronavirus, known as COVID-19 or SARS-CoV-2, causes severe acute respiratory syndrome and transmits mainly *via* respiratory secretions, droplets, or aerosols (1). Iran's first official coronavirus disease 2019 (COVID-19) case was recorded on 19 February 2020 in Qom (2). Following the declaration of COVID-19 as a pandemic by the World Health Organization, most countries announced preparedness plans to deal with COVID-19, including quarantine, social distancing, hand washing, and wearing face masks (3).

In Iran, the law on face mask uses in public places, government offices, and banks were approved on June 4, 2020. However, an observational study showed a low compliance percentage with this guideline (4). Although the Use of masks is important for controlling and preventing COVID-19 is influenced by various factors, including demographic, socioeconomic, and health behavior.

Socioeconomic status (SES) is usually measured by educational levels, income, occupation, or composite indices (5). Most epidemiological studies agree on the role of the socioeconomic gradient in health. Generally, individuals with lower socioeconomic status are more likely to die earlier and have a higher incidence of diseases than those with higher socioeconomic status (6). Also, poverty correlates with fragile health status, bringing on senior susceptibility to SARS-CoV-2 comorbidities (7). On the other hand, individuals with lower SES are more likely to be frontline workers with higher potential exposure to the virus (8). In contrast, individuals of higher SES are more likely to be working or furloughed at home with comforts such as a well-stocked pantry, stable internet, and spacious living arrangements (9).

In this study, we assessed socioeconomic factors' impact on mask use in the Ahvaz metropolitan. Also, we examined the confounder factors of age and gender in this study. Therefore, we intend to evaluate the effects of some socioeconomic indicators, including land price, literacy, and employment, on the behavior of mask use. The results can be used by health system policymakers in planning, preventing, and managing COVID-19 disease.

## Materials and methods

### Study design and setting

This study investigated the relationship between socioeconomic factors and face mask usage in Ahvaz city, southwest Iran, from August 2 to 11, 2020. Ten thousand four hundred forty pedestrians were assessed from 92 neighborhoods. An observation method was used for the individual data gathering. The findings of prevalence rates and demographic factors, besides details of the study methods, have already been published (4). Because it was impossible to collect socioeconomic data by observation method, these data were gathered as secondary data in the form of ecologic or aggregated data for each neighborhood.

The metropolitan city of Ahvaz is the capital of Khuzestan province in southwest Iran. According to the latest national census, its population is about 1,300,000. Ahvaz has a hot and usually wet climate. Low compliance rates with the protocols, including mask use and social distancing, have often been observed in the city during the COVID-19 pandemic (4). Hence, Ahvaz is among the cities with the highest incidence and mortality rates of COVID-19 in the country and usually is located in the red zone of the disease.

The Ethics Committee of Ahvaz Jundishapur University of Medical Sciences (IR.AJUMS.REC.1399.396) approved this study.

### Sample size and sampling method

We used the formula for estimating a population proportion to determine the sample size. For this purpose, α = 0.05, *p* = 0.5, d = 0.04 and a design effect equal to 1.6 were considered. A sample size of 960 is estimated for each district. We used a proportional-to-size sampling method for each district's determinant number of clusters. The final sample size needed for this study was estimated at 10,440 pedestrians. In total, 174 clusters of 60 people from 92 urban neighborhoods of Ahvaz were assessed in this study. A multistage sampling method was applied to select the participants. In the first stage, every eight urban districts were considered a stratum, and the defined number of clusters was assigned to each neighborhood proportionally to their size. Each cluster included 30 pedestrians, and the location of the observation stations was determined by a targeted sampling strategy from the busy passages of each neighborhood. At each station, data from 60 pedestrians were collected (4).

### Data collection

In this study, the data were measured on two levels: Individual-level studies collect information on outcome, exposure, and covariates for each individual. In this study, data about age, gender, and mask usage, were collected at the individual level based on the observation method. Because the observational method is one of the most valid methods in the study of behaviors, we used this method in this study. We collected the opinions of 10 experts on health sciences and social sciences as a panel expert, to validate the study checklist. To increase the validity, the same standard training for observers was used. Observers at each station, data of 60 pedestrians were recorded, including gender, approximate age, and Use of the facemask. Besides, the observers in this study have not been able to ask the exact age of the participants; therefore, approximate ages were recorded instead (4). The observation was performed during the busy hours of each area from 9.00 to 13.00 and 17.00 to 23.00. Study units in ecologic studies are groups, while partially ecologic studies use a combination of individual-level and group-level variables (10, 11). The data on exposure variables (socioeconomic indicators) were gathered at the group and neighborhood levels as secondary data. Hence the design of this study can be partially ecologic. The three main socioeconomic status factors are employment, education, and income (5). We used three socioeconomic indicators, including the average land price of neighborhoods (as a proxy of wealth status for the residences of each neighborhood), literacy rates of neighborhoods, and employment rates of neighborhoods in this study. The distribution of quantitative variables of the land price of neighborhoods, employment percentage, and literacy rates was divided into five equal categories as the quintiles. The average land price of neighborhoods was obtained from the Municipality of Ahvaz city. Moreover, the data on literacy and employment rates of the neighborhoods were obtained from The Planning and Budget Organization, based on The National Census of Population and Housing conducted in 2016.

### Statistical analysis

Mean, standard deviation, and percent have been used to describe the data. For group-level analyses, partial correlation coefficients were applied to assess the relationship between the three socioeconomic indicators and the prevalence rates of mask usage in the neighborhoods, controlled for age and gender. Partial correlation is a method used to describe the relationship between two variables while taking away the effects of another variable, or several other variables, on this relationship. For individual-level analyses, the averages of socioeconomic indicators of each neighborhood were assigned to every subject in that neighborhood. Then we used a one-way Analysis of Covariance (ANCOVA) to compare the prevalence rates of mask usage between the quintiles of the assessed socioeconomic indicators, adjusted for age and gender. Then, we used *post-hoc* Tukey to compare quintiles pair by pair. The Statistical significance level was considered <0.05. All data were analyzed with SPSS version 22.0 software.

## Results

A total of 10,440 pedestrians were observed in terms of face mask usage to collect individual-level data. The mean ± SD age of the assessed pedestrians was 32.2 ± 15.1 years. Among the observed individuals, 7,072 (67.9%) were male. The overall prevalence of face mask usage was 45.6% (95% CI, 44.6–46.5). The prevalence rates of face mask usage by demographic characteristics in the previously published article (4) Table 1.

**Table 1:** Prevalence rates of face mask usage by sex, age group, and urban district (4).

	**Number of observed pedestrians**	**Face mask usage**	
		***n***	**Prevalence (CI 95%)**
Overall prevalence	10,422	4,749	45.6 (44.6–46.5)
**Sex**			
Male	7,063	2,734	38.7 (37.6–39.9)
Female	3,336	2,009	60.2 (58.5–61.9)
**Age group**			
0–9 y	719	191	26.6 (23.4–30.0)
10–19 y	1,444	628	43.5 (40.9–46.1)
20–29 y	2,455	1,161	47.3 (45.3–49.3)
30–39 y	2,777	1,226	44.1 (42.3–46.0)
40–49 y	1,587	796	50.2 (47.7–52.6)
50–59 y	998	480	48.1 (45.0–51.2)
60–69 y	352	211	59.9 (54.6–65.1)
70 and older	60	43	71.7 (58.6–82.5)
**Urban district**			
One	1,673	809	48.4 (45.9–50.8)
Two	1,140	725	63.6 (60.7–66.4)
Three	1,377	684	49.7 (47.0–52.3)
Four	1,138	606	53.3 (50.3–56.2)
Five	960	307	32.0 (29.0–35.0)
Six	1,439	301	20.9 (18.1–22.2)
Seven	1,197	430	35.9 (33.2–38.7)
Eight	1,498	887	59.2 (56.7–61.7)

The prevalence rates and the confidence interval of 95% of the prevalence of mask use for quintiles of socio-economic have been shown in Table 2. In this analysis, three socioeconomic indicators, including Land price, Literacy rate, and the Employment rate for each neighborhood, were used to assess the effects of socioeconomic factors on face mask usage in pedestrians. Because the different numbers of pedestrians were studied in each neighborhood, the weighted prevalence rates were used in the neighborhood-level analyses. Partial correlation coefficients were applied to assess the relationship between the three socioeconomic indicators and the prevalence rates of mask usage, controlled for age and gender.

**Table 2:** Prevalence rates of face mask usage by socioeconomic factors.

**SES indicator**	**Number of observed pedestrians**	**Face mask usage**	
		***N***	**Prevalence (CI 95%)**
**Land price quintiles**			
Q1	1,380	379	27.5 (25.1–29.9)
Q2	1,253	543	43.3 (40.6–46.1)
Q3	1,920	722	37.6 (35.4–39.8)
Q4	1,917	891	46.5 (44.2–47.8)
Q5	3,714	2,154	58.0 (56.4–59.6)
**Employment quintiles**			
Q1	1,317	458	34.8 (32.2–37.4)
Q2	2,038	768	37.7 (35.6–39.8)
Q3	1,616	692	42.8 (40.4–45.3)
Q4	2,038	905	44.4 (42.2–46.6)
Q5	2,456	1,454	59.2 (57.2–61.2)
**Literacy quintiles**			
Q1	240	20	8.3 (5.2–12.6)
Q2	3,533	933	26.4 (25.0–27.9)
Q3	1,435	748	52.1 (49.5–54.7)
Q4	1,680	887	52.8 (50.4–55.2)
Q5	2,637	1,693	64.2 (62.3–66.0)

**Figure 1:**
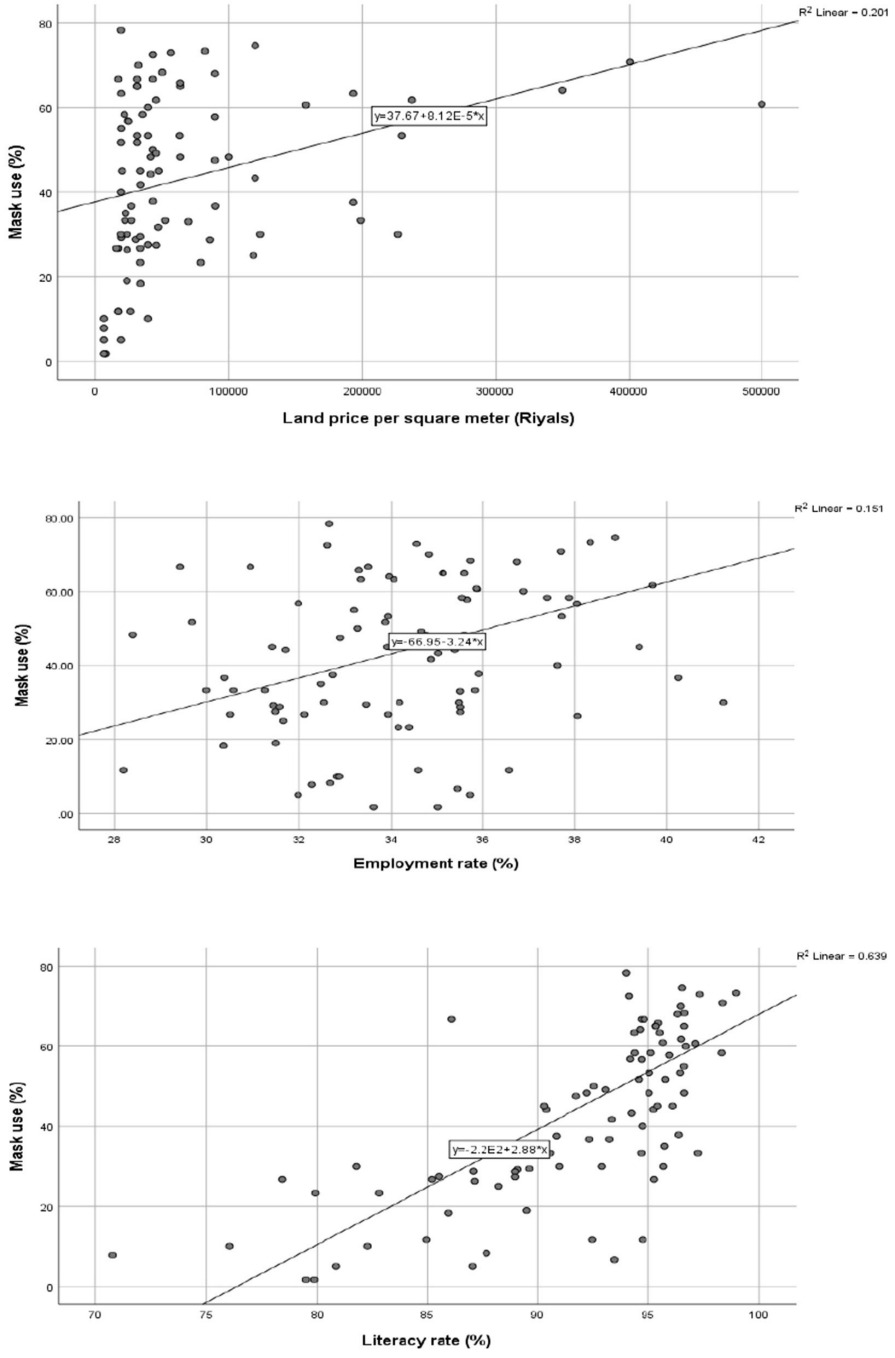
Partial correlation between the SES indicators and prevalence of mask usage at the neighborhood level, controlled for age and gender.

Our results indicated that the three assessed socioeconomic indicators were directly correlated to the prevalence of mask usage. A direct and significant correlation was seen between the average land price of neighborhoods and the prevalence of mask usage (*r* = 0.45, *p* < 0.001). A direct and significant correlation was seen between the employment rate for residents of neighborhoods and the prevalence of mask usage (*r* = 0.39, *p* < 0.001). The literacy rate for residents of neighborhoods and the prevalence of mask usage was strongly correlated (*r* = 0.78, *p* < 0.001). Also, among them, the literacy rate showed the strongest correlation with the prevalence of mask usage, while the strength of the correlations for land price and the employment rate was almost the same. The scatter diagrams are presented in Figure 1.

We used a one-way Analysis of Covariance (ANCOVA) in individual-level analysis to compare the prevalence rates of mask usage between the quintiles of the assessed socioeconomic indicators, adjusted for age and gender. Table 3 shows the adjusted prevalence rates of face mask usage by quintiles of the socioeconomic indicators with their 95% confidence intervals.

**Table 3:** Comparison of the adjusted prevalence rates of face mask-wearing among quintiles of the socioeconomic factors, using Analysis of Covariance (ANCOVA).

**Quintiles**	**Adjusted prevalence rates (CI 95%) of mask usage** ^*^		
	**Land price**	**Employment**	**Literacy**
Q1	28.6 (27.7–29.5)	39.8 (38.8–40.7)	12.4 (10.9–14.0)
Q2	46.4 (45.4–47.3)	39.0 (38.3–39.8)	27.2 (26.8–27.6)
Q3	40.1 (39.3–40.8)	42.5 (41.7–43.4)	51.5 (50.8–52.1)
Q4	45.1 (44.3–45.8)	40.3 (39.5–41.1)	52.0 (51.4–52.5)
Q5	56.0 (55.4–56.5)	59.1 (58.4–59.7)	63.6 (63.1–64.0)
*P*-value	<0.001	<0.001	<0.001

* The adjusted prevalence rates are calculated using Analysis of Covariance (ANCOVA), adjusted for age and gender.

**Figure 2:**
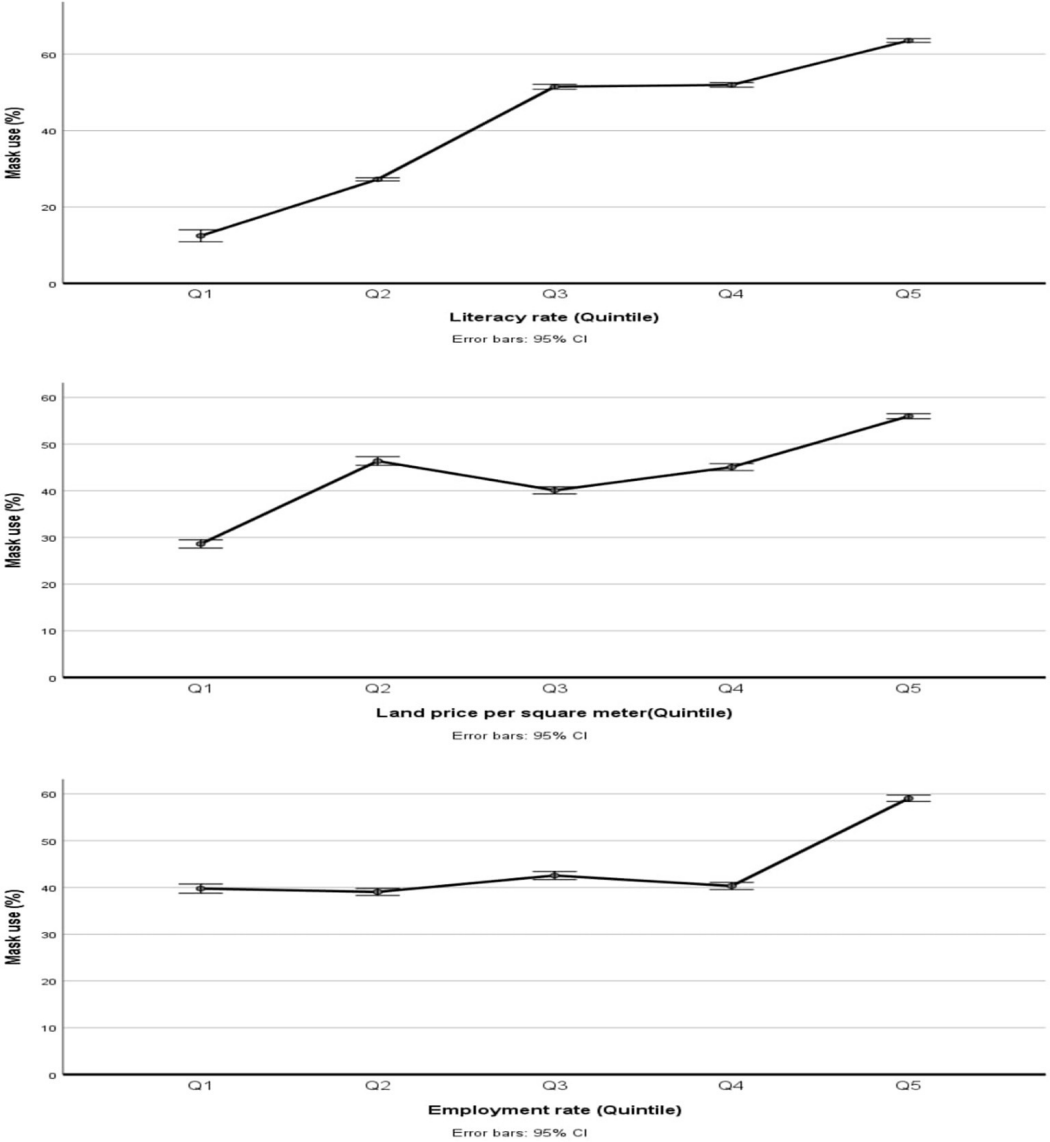
Comparison of the prevalence rates of mask usage among quintiles of the SES indicators, adjusted for age and gender.

A comparison of the adjusted prevalence rates of mask usage among quintiles of the assessed socioeconomic indicators are presented in Figure 2. The overall trend of the prevalence rates of mask usage for all three studied socioeconomic indicators increased as the participants' socioeconomic status increased.

A comparison of the prevalence rates of mask usage among the five quintiles of the land price of neighborhoods was statistically significant (*p* < 0.001). Tukey tests for pairwise comparisons showed that the prevalence rate of mask use in the Q5 group was significantly higher than in other quintiles, which means that the prevalence of mask use was higher in the residences of the richest neighborhoods. Also, the prevalence rates of Q4 and Q3 were higher than Q1 (*p* < 0.001) but the prevalence rates of Q4 and Q3 were lower than Q2 (*p* = 0.035 and *p* < 0.001, respectively).

Besides, the difference in the prevalence rates of mask usage among the five quintiles of employment rates of neighborhoods was statistically significant (*p* < 0.001). Tukey tests for pairwise comparisons indicate that the prevalence rate of mask use in Q5 was higher than the other quintiles (*p* < 0.001). Also, the rates in Q3 were higher than Q1, Q2, and Q4 (*p*<0.001). The prevalence rate in Q4 was higher than in Q2 (*p* = 0.024). However, there were no significant differences between the other pairs.

Also, a comparison of the prevalence rates of mask usage among the five quintiles of literacy rates for neighborhood residences was statistically significant (*p* < 0.001). Tukey tests for pairwise comparisons showed that as the literacy rates increased, the prevalence rates of mask use increased (*p* < 0.001) except between Q3 and Q4 (*p* = 0.3).

## Discussion

In this study, we investigated the impact of socioeconomic factors on mask use in the Ahvaz metropolitan. Socioeconomic status (SES) indicators, including education level, employment status, and household income (5), have previously been shown to be significant predictors of precautionary behaviors (12). There is a socioeconomic gradient for morbidity and mortality rates and a strong association between low income and poor health, with lower socioeconomic groups carrying a higher burden of chronic (10, 11, 13) and infectious diseases (14). Recent studies have shown that socioeconomic levels affect behaviors contributing to illness and death during the COVID-19 pandemic (15). Face mask use plays a significant role in controlling COVID-19 disease (16). Our study showed that this preventive behavior was more common in people with higher socioeconomic status.

This study demonstrated that preventive behavior was more common in people with higher socioeconomic status. These findings are in line with other studies (17, 18). However, another study indicates that no association between mask-wearing and either social and economic factors or clinical care (19).

In this study, the prevalence of mask usage in older people and women was significantly higher than in the others (*p* < 0.001). This is in line with two studies in the United States that showed wearing a mask increased significantly with age and was greater for females than males (20, 21). Another study indicates that older individuals are slightly less likely to wear face masks than younger individuals. Age did not have significant relations with any face mask perceptions (22).

We found that education is positively associated with self-protective behaviors. Along the same line, the findings showed that participants with low education, less wore masks and fewer canceled personal/social activities, and more had visitors in their residences (23). Also, education levels were positively associated with preventive behaviors regarding COVID-19, which is consistent with previous studies (24–31).

A study conducted on 5,009 American adults showed that literacy has a significant relationship with wearing a mask, but there was no significant relationship between wearing a mask and income (32). Also, the COVID-19 Health Information Survey conducted among adults in Hong Kong showed that the difference in the eHEALS score between participants in the highest and the lowest SES was higher for education than income (33). These findings were consistent with the results of our study, so among the three assessed socioeconomic indicators in our research, literacy was the most correlated indicator to mask usage.

Besides, the findings of our study showed that with the increase in the employment percentage of people in neighborhoods, face mask use has also increased. This result has been confirmed by the finding of another study that having a job was related to a greater likelihood of wearing a mask because the people who perceive their risk to be high tend to engage in more preventive and fewer risky behaviors (23).

Also, the results of our study showed that face mask use was higher in areas with higher land prices, which is a proxy of socioeconomic status. Previous studies showed that rates of infectious diseases are associated with poverty rates (34–36). Such association is because poorer communities are often less supplied to deal with the health and financial consequences of COVID-19 (37). Also, the link between the incidence of COVID-19 with lower income and lower SES is most likely due to the overall economic conditions such as poverty, performing essential public tasks, poor quality, and overcrowded housing, as well as an obligation to use public transport (8). These findings are similar to an ecological study conducted in the United States of America (USA) that points out a higher percentage of COVID-19 cases in areas with lower income and higher poverty levels (37). As the COVID-19 pandemic has progressed, it has become clear that there are many inequalities in the susceptibility and severity of the disease (38). Some studies reported associations between income and COVID-19 outcomes (39–41). Those with lower income may be disadvantaged in adopting preventive pandemic reactions and take more risky behaviors out of necessity due to their lower socioeconomic status and associated occupational status and need to work and use public transportation (42). In line with our study, a study in the USA demonstrated that people with the highest income status wore face masks greater than others (43) on the other hand, economic position is also associated with levels of trust in social institutions, including the healthcare system (44).

The study's strength was that a large sample size led to the precise estimation of the rates. It can be found in the narrow confidence intervals. Our study had several limitations: Due to the data gathering being based on observation, we could not collect data about participants' socioeconomic status. Hence, we used group-level data instead of individual-level data. This, in turn, can produce an Ecologic fallacy. Besides, it was impossible to distinguish the exact age of the subjects, so approximate ages were recorded instead. Accordingly, a non-differential misclassification in a grouping of the ages may have occurred.

## Conclusion

In many countries, there are some differences in health behaviors and long-term health outcomes among people with different socioeconomic positions (6). Our study showed that low socioeconomic status reduces the health behavior of wearing a mask. Among the three assessed SES indicators, literacy showed the strongest correlation with the prevalence of mask usage compared to the land price and employment rate. This finding demonstrated the important role of people's education level and health literacy in implementing preventive behaviors in communities. Hence, it can be concluded that the social component of socioeconomic status has a greater effect on mask usage by people than the economic component of socioeconomic status. We proposed to improve preventive behaviors in vulnerable groups, including less-educated and low-income people, educational interventions such as making videos and training clips, and supportive measures such as supplying masks, soap, and hand sanitizers should be considered during the COVID-19 pandemic.

## Data availability statement

The original contributions presented in the study are included in the article/Supplementary material, further inquiries can be directed to the corresponding author.

## Ethics statement

The studies involving human participants were reviewed and approved by the Ethics Committee of Ahvaz Jundishapur University of Medical Sciences (IR.AJUMS.REC.1399.396) confirmed the morality and ethics of the study. Because the data collection method was observation and there were no human participants in the current study, obtaining informed consent is deemed unnecessary according to regulations. Written informed consent from the participants' legal guardian/next of kin was not required to participate in this study in accordance with the national legislation and the institutional requirements.

## Author contributions

BC and ZR were the study's principal investigators and drafted the manuscript. GS, MA, and MM were advisors of the study. BC performed the statistical analysis. All authors contributed to the design and data analysis and assisted in the preparation of the final version of the manuscript. All authors read and approved the final version of the manuscript.

## Funding

The Vice-Chancellor provided financial support for Research at Ahvaz Jundishapur University of Medical Sciences, Grant number U-99144. As our funding body, the Vice-Chancellor for Research at Ahvaz Jundishapur University of Medical Sciences played no role in the design of the study and collection, analysis, and interpretation of data and the writing of the manuscript.

## Conflict of interest

The authors declare that the research was conducted in the absence of any commercial or financial relationships that could be construed as a potential conflict of interest.

## Publisher's note

All claims expressed in this article are solely those of the authors and do not necessarily represent those of their affiliated organizations, or those of the publisher, the editors and the reviewers. Any product that may be evaluated in this article, or claim that may be made by its manufacturer, is not guaranteed or endorsed by the publisher.
